# First epidemiological report of feline heartworm infection in the Barcelona metropolitan area (Spain)

**DOI:** 10.1186/s13071-014-0506-6

**Published:** 2014-11-12

**Authors:** José Alberto Montoya-Alonso, Elena Carretón, Laín García-Guasch, Jordi Expósito, Belén Armario, Rodrigo Morchón, Fernando Simón

**Affiliations:** Internal Medicine, Faculty of Veterinary Medicine, University of Las Palmas de Gran Canaria, 35413 Arucas, Las Palmas Spain; Cardiology Service, Hospital Veterinari Molins, 08620 Barcelona, Spain; Hospital Vet’s Avinguda, 08203 Sabadell, Barcelona Spain; Laboratory of Parasitology, Faculty of Pharmacy and Institute of Biomedical Research of Salamanca (IBSAL), University of Salamanca, Salamanca, Spain

**Keywords:** Feline, Heartworm, *Dirofilaria immitis*, Prevalence, Epidemiology, Spain

## Abstract

**Background:**

The metropolitan area of Barcelona is the most densely populated metropolitan area on the Mediterranean coast. Several studies have reported the presence of canine heartworm disease in this region; however, there are no published epidemiological data regarding feline heartworm in this region and the prevalence in this species remains unknown.

**Methods:**

Serum samples from 758 cats living in the metropolitan area of Barcelona (Spain) were collected between 2012 and 2013. To establish the seroprevalence of heartworm infection in cats, serological techniques for anti-*D.immitis* and anti-*Wolbachia* antibody detection were used while a commercial ELISA test kit was used to detect circulating *D.immitis* antigens.

**Results:**

Of these samples, 11.47% were positive to *D.immitis* and *Wolbachia* surface protein antibodies and 0.26% were positive to *D.immitis* antigens. The higher antibody seroprevalences were found in the areas that follow the courses of the rivers Llobregat and Anoia (Baix Llobregat 11.5%, Vallés Occidental 13.2%; Barcelonés 11.7%) where humidity and vegetation favour the development of the mosquito vectors. High antibody seroprevalences were also found in the urban areas (Barcelona city 13.1%; Sabadell 15.5%), which demonstrates that city cats are also at risk from *D.immitis* infection.

**Conclusions:**

Generally, in Spain cats do not receive prophylactic treatment and therefore the risk of infection is higher in this species than in dogs. Adequate prophylactic plans should be implemented in the feline population. This is the first epidemiologic study on feline heartworm infection to be carried out in continental Spain.

## Background

*Dirofilaria immitis* (heartworm) is a vector-borne zoonotic parasite. Transmission occurs through different species of culicid mosquitoes belonging to the genera *Culex*, *Aedes* and *Anopheles*, among others [1]. The domestic dog is the main biological reservoir, although infection has been reported in other carnivores, including wild canines as well as domestic and wild felids [2]. Furthermore, canine dirofilariosis constitutes a risk for the human population living in endemic areas, since *D. immitis* is the causative agent of pulmonary dirofilariosis characterized by the development of benign pulmonary nodules that mimic malignant pulmonary nodules [3].

Cats are inherently resistant to heartworm infection. This is reflected by relatively low adult worm burden, the lack of microfilaremia and their short life span, which complicates the diagnosis of this disease in the feline patient. The non-specific clinical signs, generally of a respiratory or digestive nature, are usually confused with other diseases, as well as the frequent asymptomatic course of feline dirofilariosis in which the sudden death of the animal is sometimes the only clinical sign shown All the afore mentioned contribute to the difficulty of the diagnosis in this species [4].

Recently feline heartworm disease has been increasingly diagnosed due to heightened awareness of the disease in cats, improved diagnostic methods and the increasing number of cases reported in the last few years. It is now generally accepted that heartworm disease may occur in cats in any area where dogs are infected, with a prevalence rate of infections in cats between 5% and 20% of that for dogs in the same geographical area [5,6].

In Europe, heartworm disease is endemic in Mediterranean countries [7,8], although some studies have shown that it is spreading towards central and eastern areas of the European continent which were previously considered free of the disease [9,10]. Spain is an endemic country and the highest prevalences have been reported in the Mediterranean coast and Southern Spain, along the shores of major rivers, estuaries and marshlands, as well as in the Canary Islands [10,11]. The metropolitan area of Barcelona is the most densely populated metropolitan area on the Mediterranean coast, with a population above 5 million. Several studies have reported the presence of canine heartworm disease in this region, ranging from 0.6% [12] and 1.2% [13] to 3.57% [14]. The last study in 2006 reported a canine prevalence of 2% [15].

Despite the numerous studies that have been carried out in canine heartworm, there are no published epidemiological data regarding feline heartworm in this region. In fact, no data on feline heartworm in continental Spain have been published to the authors’ knowledge. So far, this subject has only been studied in the Canary Islands [11]. The aim of this study was to determine the prevalence of *D. immitis* in cats from the metropolitan area of Barcelona.

## Methods

The present study included 758 client-owned cats presented to veterinary clinics. Of these, 56.5% were males and 43.5% were females. By breed, 77.2% were domestic shorthair, 12.3% were Persian, 6.7% were Siamese and 3.8% other breeds. The age ranged from 6 months to 18 years, with a mean range of 6.8 years. 56% of the cats were indoor (cats always kept inside the house), 28% was outdoor (those always kept outside the house) and 16% for cats that spent at least 1–50% of their time outdoors (indoor/outdoor).

**Figure 1:**
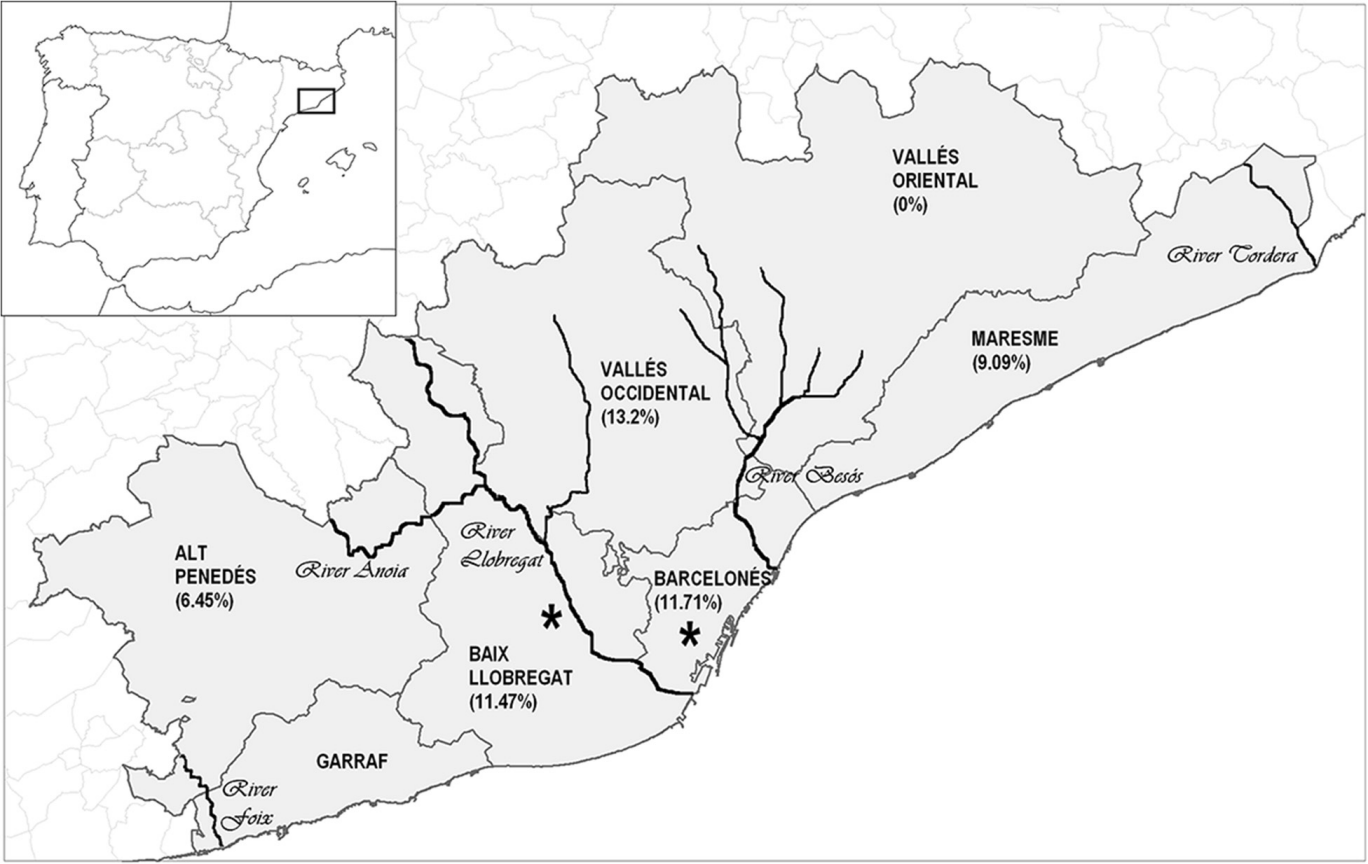
**Feline** ***Dirofilaria immitis*** **antibody seroprevalence (%) in the Barcelona metropolitan area (Spain) by regions: Alt Penedès, Baix Llobregat, Barcelonès, Garraf (not included in the present study), Maresme, Vallés Occidental and Vallés Oriental.** The main rivers are illustrated as black lines. The asterisks (*) indicate the location of the antigen-positive cats.

The samples were collected at 27 veterinary centres located in the studied area, from cats presented to veterinary clinics between September 2012 and September 2013. The animals lived in 6 of the 7 regions which form the metropolitan area of Barcelona: Alt Penedés, Baix Llobregat, Barcelonés, Maresme, Vallés Occidental and Vallés Oriental (Figure 1).

The criteria for inclusion of cats were cats over 6 months of age, not having travelled outside the area of interest of the present study, never having received treatment for heartworm disease, no previous history of heartworm infection, and owner consensus to participate in the survey. A complete record was kept for each animal, including identification (age, sex, and breed), clinical history, and demographic data. All owners gave their consent to participate in the study. The study was approved by the ethical committee of Veterinary Medicine Service of Las Palmas de Gran Canaria University and was carried out in accordance with the current European legislation on animal protection.

Blood samples were collected from the cephalic or jugular vein, placed in 3 ml serum tubes and centrifuged. Serum was kept at –20°C until tests were performed. To establish the seroprevalence of heartworm infection in cats, serological techniques for anti-*D.immitis* and anti-*Wolbachia* antibody detection were used, as described by Morchón *et al*. [16] with some modifications. In brief, the plates were coated with 0.8 μg of *D. immitis* somatic antigen and *Wolbachia* surface protein (WSP). Serum samples were prepared at 1/100 for anti-*D.immitis* serum antibodies and 1/40 for anti-WSP antibody detection. Anti-feline IgG antibody, horseradish peroxidase-labelled (Kirkegaard and Perry Laboratories, Gaithersburg, MD, USA), was applied at 1/4000 dilution. The optical densities were measured in an Easy-Reader (Bio-Rad Laboratories, Hercules, CA, USA) at 492 nm. Cut-off points of ELISA *D. immitis* 0.8 and ELISA WSP 0.6 were obtained as arithmetic mean optical density ±3 standard deviations of serum of clinically healthy cats. The cats were considered seropositive when anti-*D.immitis* and anti-WSP antibodies presented jointly [11,17].

All feline serum samples were tested for circulating *D.immitis* antigens using a commercial immunochromatographic test kit (Uranotest Dirofilaria®, Urano Vet SL, Barcelona, Spain) according to manufacturer’s instructions.

Data were analyzed using SPSS Base 20.0 software for Windows (SPSS Inc./IBM, Chicago, IL, USA). Descriptive analysis of the considered variables was carried out considering the proportions of the qualitative variables. *χ*2 and Fischer exact tests to compare proportions were performed. In all cases, the significance level was established at p < 0.05.

## Results

11.47% (87/758) of cats showed *D.immitis* and WSP antibody seropositivity, of these 62.1% were males and 37.9% were females (p < 0.001)., Seroprevalence by age was 8.1% (<1 year), 9.7% (1-3 years), 13.7% (3-6 years), 13% (6-9 years), 9.5% (9-12 years), 10.8% (12-15 years) and 12% (>15 years), with no significant differences between groups. By breed, the higher prevalences were observed in Siamese cats (19.6%), mixed breed cats (18.2%) and domestic shorthair cats (11.3%), followed by Persian cats (9.7%), while the seroprevalence in the other studied breeds was 0%. Seroprevalence for outdoor cats was 21.2%, compared with 7.05% for indoor cats and 9.9% for indoor/outdoor cats (p < 0.001). Of the seropositive cats, 24.1% (21/87) showed respiratory signs (p < 0.001). By regions, the higher seroprevalences were found in cats living in Vallés Occidental 13.2% (14/106), Barcelonés 11.7% (28/239) and Baix Llobregat 11.5% (42/366). Lower seroprevalences were found in Maresme 9.1% (1/11) and Alt Penedés 6.4% (2/31). Vallés Oriental showed a seroprevalence of 0% (0/5). There were no statistically significant differences between regions except Vallés Oriental. In Sabadell (capital of Vallés Occidental) 15.5% of the cats studied were seropositive (9/58) (p < 0.001) while in the urban area of Barcelona (located within Barcelonés) there were 13.1% seropositive cats (16/199) (p < 0.001).

0.26% (2/758) of the cats tested positive to *D.immitis* antigens. Of the 2 cats that tested positive, one lived in the urban area of Barcelona (Barcelonés) and the other one in Baix Llobregat. Both cats were outdoors and tested positive to antibodies tests.

## Discussion

The present study is the first epidemiological report of seroprevalence of *D. immitis* in cats in Barcelona and in continental Spain, previously only having been reported in the Canary Islands and in the North of Portugal [11,17]. 11.47% of cats were positive to antibodies tests; these detect antibodies produced by the host in response to infection and become positive after 2-3 months from infection, in the prepatent phase; however, they can remain positive for a long time after the death of the parasite and therefore do not differentiate a current or a past infection [4,18]. Antibody tests are suitable to assess the infection risk within a feline population and cat antigen negative and antibody positive should be subject to additional diagnostic tests, such as echocardiography and thoracic X-ray [18]. Of the seropositive cats 24.1% showed respiratory signs, which may be a vascular and parenchymal inflammatory response associated with the arrival and death of *D. immitis* in the distal pulmonary arteries in cats, this has been previously reported in seropositive cats from Barcelona [4,19,20].

Only 0.26% of cats were positive to *D.immitis* antigens. The sensibility of antigen testing is relatively low in cats, and because these tests only detect adult and female worms a negative result does not rule out an infection from male worms or pre-adult worms, most of which are common in cats [4]. Furthermore, the test may be negative in some patients with an infection from a single female adult worm [21,22]. Hence, it is recommended carry out both antigens and antibodies tests be carried out in this species [21]. It is estimated that the feline infection is 5-20% of that of the canine population in the same area. Venco et al. [6] reported a feline prevalence of approximately 10% of the prevalence in dogs in an endemic area in Italy. Preliminary results in canine heartworm infection found by our research team in the same area of study and timeframe, place the canine prevalence at about 2.5% [unpublished data], placing a feline prevalence of 10.4% of that in dogs; therefore, the heartworm prevalence found in the cats of the metropolitan area of Barcelona may be considered within normal ranges when compared to canine prevalence.

The particular climatic conditions (Mediterranean climate) in Barcelona make it an endemic area for heartworm. These conditions are favourable for the development of the mosquito vectors. The results showed that the higher antibody seroprevalences are mainly found following the courses of rivers and streams (Figure 1), where vegetation and humidity are higher than in other points of the metropolitan area of Barcelona and correspond to irrigated crops, as well as rural areas where cats live generally outdoors. In the results, one of the cats that tested positive to the antigen test lived in the Baix Llobregat region, an agricultural and fluvial area near Barcelona, where a previous study reported a prevalence of canine heartworm of 12.8% [23].

Meanwhile, higher antibody seroprevalences were present in the urban areas of Vallés Occidental (Sabadell, 15.5%) and Barcelonés (Barcelona city, 13.06%). Furthermore, one of the cats that tested positive to the antigen test lived in the urban area of Barcelona, close to an urban park. These results demonstrate that city cats are also at risk from heartworm infection. It has been described that building construction and human activity increase the density of potential hosts and develop a suitable environment for the proliferation of certain species of mosquitoes due to an increase in the provision of water sources and vegetation [24,25]. Besides, urban sprawl has led to the formation of “heat islands”, as buildings retain heat during the day and which is subsequently radiated during the night. This can potentially create microenvironments that support the development of heartworm larvae in mosquito vectors during colder months, thus lengthening the transmission season [10,26].

A higher rate of positivity for *D.immitis* in cats living outdoor was observed when compared with cats living indoor or outdoor/indoor. Although an indoor lifestyle does not protect cats from infection, a higher prevalence is to be expected in outdoor cats, and our results are similar to those reported in previous studies [5].

## Conclusions

In the light of previous studies carried out in canine heartworm disease, with a prevalence of 2-2.5% [15; unpublished data], the feline population is at risk from heartworm infection in the metropolitan area of Barcelona. Furthermore, cats do not usually receive prophylactic treatment therefore the risk of heartworm infection is higher in this species than in dogs. The implementation of adequate prophylactic plans in cats from this region should be carried out as well as the development of further research to evaluate the progress of the epidemiology in this species.
